# Investigating the Safety and Efficacy of Therapeutic Hypothermia in Pediatric Severe Traumatic Brain Injury: A Systematic Review and Meta-Analysis

**DOI:** 10.3390/children11060701

**Published:** 2024-06-07

**Authors:** Seyed Ahmd Naseri Alavi, Mohammad Amin Habibi, Alireza Majdi, Bardia Hajikarimloo, Farhang Rashidi, Sahar Fathi Tavani, Poriya Minaee, Seyed Mohammad Eazi, Andrew J. Kobets

**Affiliations:** 1 Department of Neurosurgery, School of Medicine, Emory University, Atlanta, GA 30033, USA; 2 Department of Neurosurgery, Shariati Hospital, Tehran University of Medical Sciences, Tehran 14399, Iran; 3 Research Group Experimental Oto-Rhino-Laryngology, Department of Neuroscience, Leuven Brain Institute, KU Leuven, 3000 Leuven, Belgium; 4 Department of Neurosurgery, Shohada Tajjrish Hospital, Shahid Beheshti University of Medical Science, Tehran 14399, Iran; 5 School of Medicine, Tehran University of Medical Sciences, Tehran 14399, Iran; 6 Student Research Committee, Faculty of Medicine, Qom University of Medical Sciences, Qom 999067, Iran; 7 Department of Neurological Surgery, Montefiore Medical Center and the Albert Einstein College of Medicine, Bronx, NY 10467, USA

**Keywords:** pediatric traumatic brain injury, hypothermia, systematic review, meta-analysis

## Abstract

Background: Prior guidelines recommended maintaining normothermia following traumatic brain injury (TBI), but recent studies suggest therapeutic hypothermia as a viable option in pediatric cases. However, some others demonstrated a higher mortality rate. Hence, the impact of hypothermia on neurological symptoms and overall survival remains contentious. Methods: We conducted a systematic review and meta-analysis to evaluate the effects of hypothermia on neurological outcomes in pediatric TBI patients. The PubMed/Medline, Scopus, and Web of Science databases were searched until 1 January 2024 and data were analyzed using appropriate statistical methods. Results: A total of eight studies, comprising nine reports, were included in this analysis. Our meta-analysis did not reveal significant differences in mortality (RR = 1.58; 95% CI = 0.89–2.82, *p* = 0.055), infection (RR = 0.95: 95% CI = 0.79–1.1, *p* = 0.6), arrhythmia (RR = 2.85: 95% CI = 0.88–9.2, *p* = 0.08), hypotension (RR = 1.54: 95% CI = 0.91–2.6, *p* = 0.10), intracranial pressure (SMD = 5.07: 95% CI = −4.6–14.8, *p* = 0.30), hospital length of stay (SMD = 0.10; 95% CI = −0.13–0.3, *p* = 0.39), pediatric intensive care unit length of stay (SMD = 0.04; 95% CI = −0.19–0.28, *p* = 0.71), hemorrhage (RR = 0.86; 95% CI = 0.34–2.13, *p* = 0.75), cerebral perfusion pressure (SMD = 0.158: 95% CI = 0.11–0.13, *p* = 0.172), prothrombin time (SMD = 0.425; 95% CI = −0.037–0.886, *p* = 0.07), and partial thromboplastin time (SMD = 0.386; 95% CI = −0.074–0.847, *p* = 0.10) between the hypothermic and non-hypothermic groups. However, the heart rate was significantly lower in the hypothermic group (−1.523 SMD = −1.523: 95% CI = −1.81–−1.22 *p* < 0.001). Conclusions: Our findings challenge the effectiveness of therapeutic hypothermia in pediatric TBI cases. Despite expectations, it did not significantly improve key clinical outcomes. This prompts a critical re-evaluation of hypothermia’s role as a standard intervention in pediatric TBI treatment.

## 1. Introduction

Traumatic brain injury (TBI) refers to a physiological disruption within the brain resulting from external forces, which can manifest as either penetrating or non-penetrating trauma. This condition encompasses a spectrum of symptoms, including alterations in consciousness, seizures, coma, and, in extreme cases, death [1,2]. Certain symptoms of concussion may manifest immediately following the injury, while others may develop gradually over time. Additionally, certain symptoms can persist for an extended duration, potentially becoming chronic or long-lasting [3]. TBI exhibits three distinct peaks in incidence: the first occurs in infants and children up to 4 years old, the second is observed in adolescents and young adults aged between 15 and 24, and the final peak occurs among individuals over 65 years old [4]. Among all age cohorts, young children exhibit one of the highest rates of emergency department visits attributable to TBI [5].

Hypothermia has demonstrated neuroprotective properties in animal studies by reducing cerebral oxygen and glucose consumption, mitigating oxidative stress, and potentially improving TBI outcomes [6,7,8,9]. While earlier guidelines advocated for maintaining normothermia due to limited evidence supporting hypothermia’s efficacy [10,11], recent research has highlighted the potential benefits of therapeutic cooling in infants and children following severe TBI [12]. Despite its promising neuroprotective effects, the precise impact of hypothermia on TBI patient outcomes, including neurological symptoms and overall survival, remains a subject of ongoing debate [13].

A prior meta-analysis of randomized controlled trials involving pediatric TBI patients found no significant differences except for the Glasgow Outcome Scale (GOS) scores [14]. Conversely, a recent meta-analysis focusing on children with severe TBI reported a notable reduction in overall mortality with therapeutic hypothermia, but no significant improvements were observed in terms of adverse outcomes, duration of pediatric intensive care unit (PICU) stay, infection rates, or arrhythmia incidence [15]. Given the potential risks associated with hypothermia and the mixed findings from previous studies, it is currently regarded as a second-line therapy for TBI [10,16,17].

As mentioned above, TBI is a significant public health concern, particularly in the pediatric population, where it is a leading cause of mortality and morbidity [18]. In addition, therapeutic hypothermia has emerged as a potential neuroprotective strategy in the management of TBI, to mitigate secondary injury mechanisms and improve patient outcomes [19]. However, the efficacy and safety of hypothermia in pediatric TBI remain subjects of ongoing debate, warranting comprehensive evaluation through systematic reviews and meta-analyses [20]. Given the ongoing controversy in the literature, the objective of this study is to systematically assess the safety and efficacy of hypothermia as a viable treatment option for TBI in children with severe TBI.

## 2. Methods

This study was designed and prepared according to the Preferred Reporting Items for Systematic Reviews and Meta-Analyses (PRISMA) statement [21].

### 2.1. Databases and Search Strategy

The electronic databases PubMed/Medline, Scopus, and Web of Science were comprehensively searched until 1 January 2024, without any language, study type, or publication year filters. The search strategy employed specific syntax tailored for each database, incorporating the keywords “Hypothermia” AND “Brain Injury” OR “Traumatic Brain Injury” OR “Brain Trauma” OR “TBI” AND “Children” OR “Pediatric”.

### 2.2. Eligibility Criteria

For the present study, a PICO (Population, Intervention, Comparison, Outcome) outline was employed to ensure precise targeting of the study aim. The study’s inclusion criteria encompassed English studies including randomized controlled trials, prospective and retrospective cohort studies, case–control studies, and cross-sectional studies examining the outcomes of hypothermia on pediatric individuals with TBI. The exclusion criteria comprised all studies other than English-language studies, and animal studies including review articles, protocol articles, and letters to the editor, and were omitted from consideration.

#### Screening Process and Data Extraction

Upon the retrieval of records from each database, they were promptly imported into EndNote V.20 software for the study selection process. Two reviewers meticulously evaluated the studies through a two-step title/abstract screening process followed by a full-text assessment. After removing duplicates, articles were selected based on the eligibility criteria. Only the most relevant articles meeting the eligibility criteria proceeded to further evaluation during the title/abstract screening. Studies meeting the inclusion criteria were chosen for statistical analysis and data extraction during the full-text assessment. Two reviewers independently conducted the screening and data extraction using a pre-designed Excel sheet, with EndNote software utilized to prevent duplication of references.

### 2.3. Statistical Analysis

All statistical analyses were performed using Comprehensive Meta-Analysis Software (CMA) version 4. The appropriate effect size measures were selected by the Cochrane Handbook. The risk ratios (RRs) were calculated for mortality, infection, arrhythmia, and hypotension rate, along with their respective 95% confidence intervals (CIs). Additionally, the standard mean difference (SMD) was calculated for intracranial pressure (ICP), length of hospital stay, and length of ICU stay between the hypothermia and control groups. Significant heterogeneity was defined as a Chi-square *p*-value of ≤0.05 and an *I*^2^ metric of >50%. Due to the high heterogeneity of the data, a random effect model was used to analyze the findings. Sensitivity analysis was conducted using leave-one-out analysis to assess the robustness of outcomes. Publication bias was assessed through visualization of the funnel plot and Egger’s test.

## 3. Results

### 3.1. Study Selection

**Figure 1:**
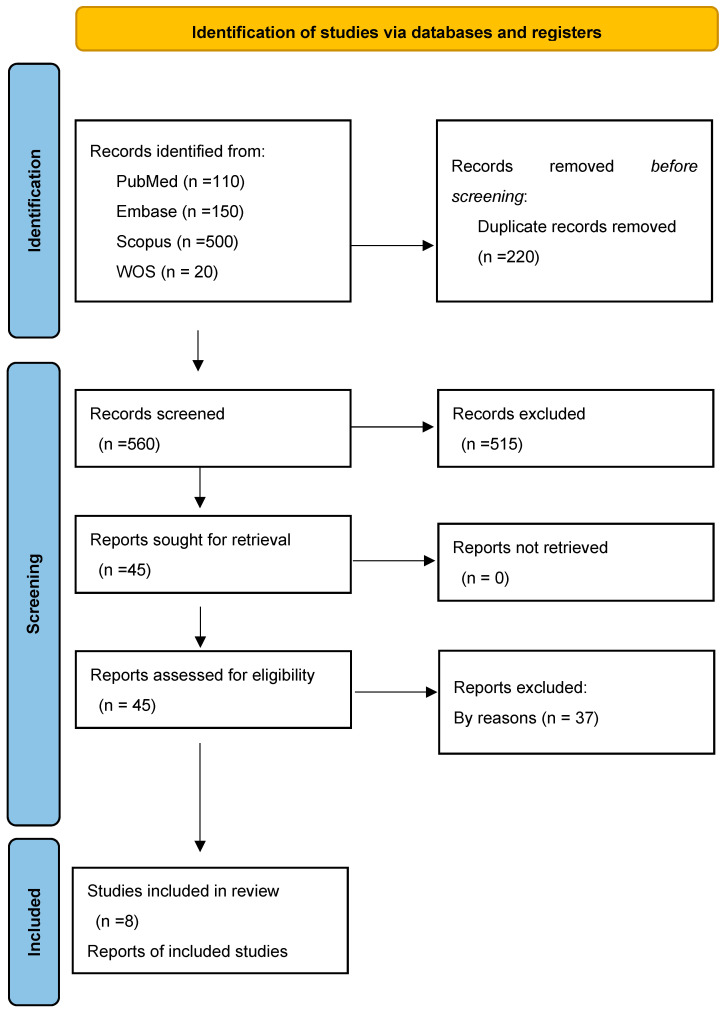
PRISMA flowchart of the study selection process.

A total of 780 records were retrieved from PubMed, Embase, Scopus, and Web of Science. Following the removal of duplicates, the abstracts from 560 articles underwent eligibility screening. Among these, the abstracts from 45 studies met the inclusion criteria, leading to the evaluation of their full-text articles. Subsequently, 37 studies were excluded during the full-text assessment. Ultimately, after thorough evaluation, eight studies (Figure 1) involving 516 participants met the inclusion criteria and were included in the analysis.

### 3.2. Study Characteristics

Table 1 presents the detailed characteristics of the included studies. The majority of studies were conducted in the United States (n = 4). Among the eight included studies, five adopted a multi-center approach, while the remaining three were single-center studies. All studies were prospectively conducted. All studies focused on individuals with a Glasgow Coma Scale (GCS) below eight (severe TBI). Rectal temperature was the primary site of temperature assessment in most studies (n = 5), followed by esophageal temperature (n = 3), as detailed in Table 2.

### 3.3. Meta-Analysis of Data

**Figure 2:**
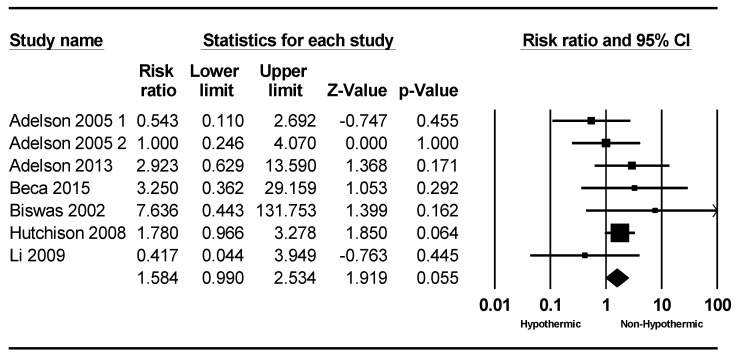
Forest plot depicting the meta-analysis of mortality rates comparing hypothermic and non-hypothermic groups in pediatric traumatic brain injury patients [22,23,24,25,27,29].

The outcomes of patients in both the hypothermia and control groups are presented in Table 3 and Table 4, respectively. A total of six studies were included in the meta-analysis of mortality, with mortality rates ranging from 0% to 30%. The meta-analysis indicated a slightly lower mortality rate in the non-hypothermic group compared to the hypothermic group, although this difference was not statistically significant (RR = 1.58; 95% CI = 0.89–2.82, *p* = 0.055), as depicted in Figure 2.

**Figure 3:**
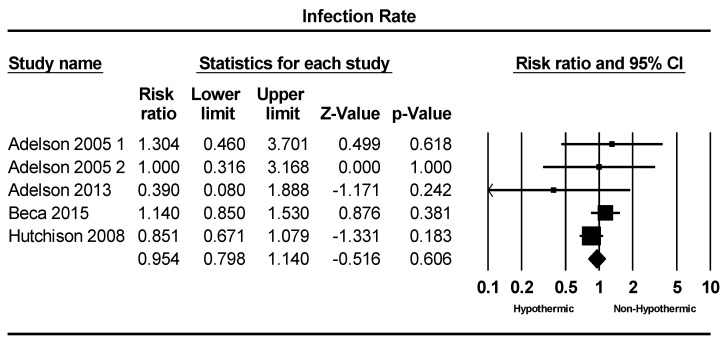
Forest plot depicting the meta-analysis of infection rate comparing hypothermic and non-hypothermic groups in pediatric traumatic brain injury patients [22,23,24,25].

A total of five studies were included in the analysis of infection incidence. Infection rates ranged from 13% to 83%. The meta-analysis revealed a slightly lower infection rate in the hypothermic group compared to the non-hypothermic group; however, this difference was not statistically significant, as illustrated in Figure 3 (RR = 0.95: 95% CI = 0.79–1.1, *p* = 0.6).

**Figure 4:**
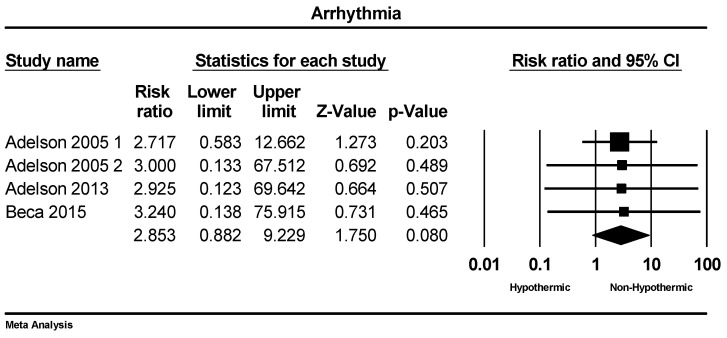
Forest plot depicting the meta-analysis of arrhythmia rate comparing hypothermic and non-hypothermic groups in pediatric traumatic brain injury patients [22,23,25].

A total of four studies were included in the meta-analysis of arrhythmia incidence. The rate of arrhythmia incidence ranged from 0% to 22% across the studies. The meta-analysis indicated a trend toward a higher incidence of arrhythmias in the hypothermic group; however, this difference did not reach statistical significance (RR = 2.85: 95% CI = 0.88–9.2, *p* = 0.08), as shown in Figure 4.

**Figure 5:**
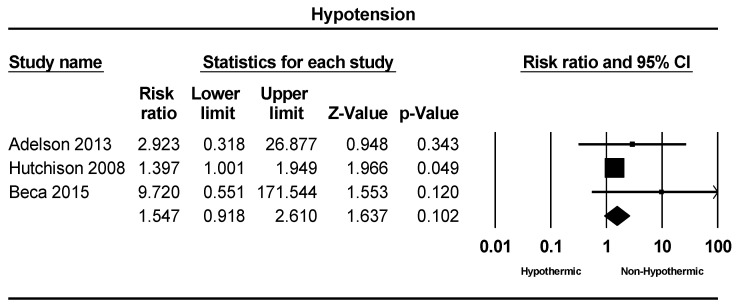
Forest plot depicting the meta-analysis of hypotension rate comparing hypothermic and non-hypothermic groups in pediatric traumatic brain injury patients [23,24,25].

A total of three studies were included in the meta-analysis of hypotension incidence. The rate of hypotension incidence varied from 0% to 45% across the included studies. The meta-analysis indicated a slightly higher incidence of hypotension in the hypothermic group compared to the non-hypothermic group; however, this difference did not reach statistical significance (RR = 1.54: 95% CI = 0.91–2.6, *p* = 0.10), as depicted in Figure 5.

**Figure 6:**
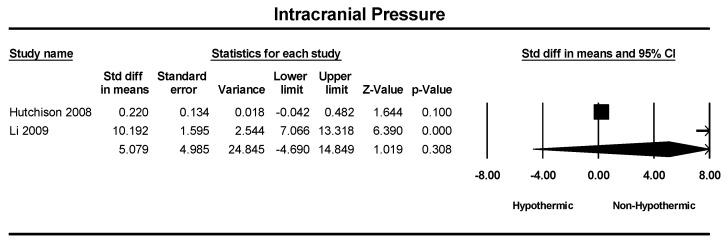
Forest plot depicting the meta-analysis of intracranial pressure comparing hypothermic and non-hypothermic groups in pediatric traumatic brain injury patients [24,29].

A total of two studies were included in a meta-analysis of intracranial pressure (ICP). The standard difference in means between the hypothermic and non-hypothermic groups was 5.07 mmHg; however, the difference was insignificant (SMD = 5.07: 95% CI = −4.6–14.8, *p* = 0.30) (Figure 6).

**Figure 7:**
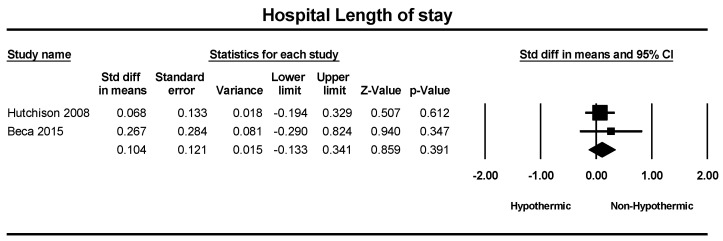
Forest plot depicting the meta-analysis of hospital length of stay comparing hypothermic and non-hypothermic groups in pediatric traumatic brain injury patients [24,25].

**Figure 8:**
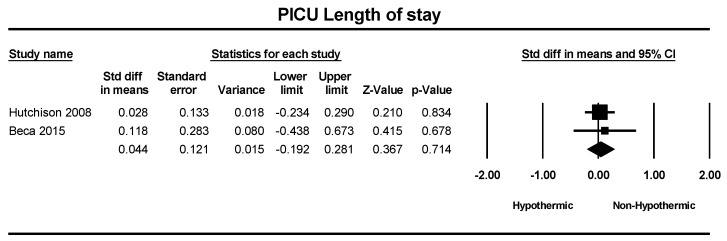
Forest plot depicting the meta-analysis of PICU length of stay comparing hypothermic and non-hypothermic groups in pediatric traumatic brain injury patients [24,25].

A total of two studies were included in a meta-analysis of hospital length of stay and pediatric intensive care unit (PICU) length of stay. The standard difference in means for hospital length of stay between groups was 0.10 days, which was not statistically significant (SMD = 0.10; 95% CI = −0.13–0.3, *p* = 0.39), as shown in Figure 7. Similarly, the standard difference in means for PICU length of stay was 0.04 days, and no statistically significant difference was observed (SMD = 0.04; 95% CI = −0.19–0.28, *p* = 0.71), as illustrated in Figure 8.

**Figure 9:**
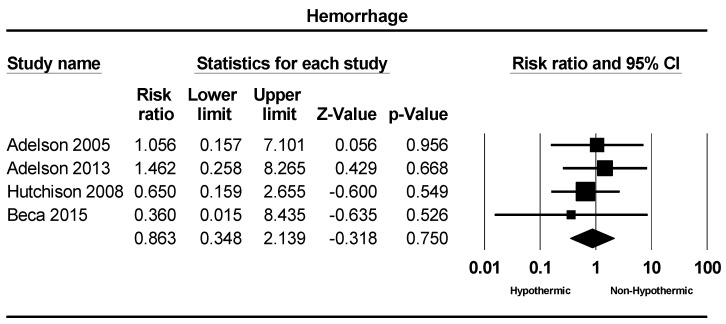
Forest plot depicting the meta-analysis of hemorrhage incidence comparing hypothermic and non-hypothermic groups in pediatric traumatic brain injury patients [22,23,24,25].

Four studies were included in a meta-analysis of hemorrhage incidence. Hemorrhage was reported in 0% to 8% of individuals across the studies. The meta-analysis indicated a slightly lower occurrence of hemorrhage in the hypothermic group compared to the non-hypothermic group; however, this difference was not statistically significant (RR = 0.86; 95% CI = 0.34–2.13, *p* = 0.75), as depicted in Figure 9.

**Figure 10:**
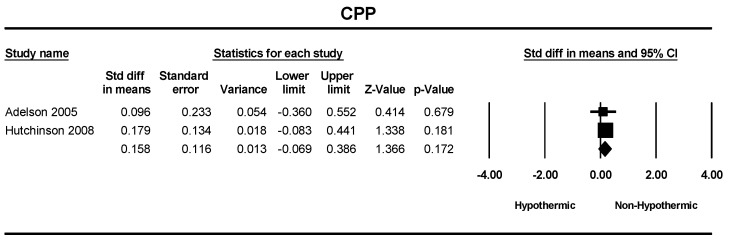
Forest plot depicting the meta-analysis of central perfusion pressure comparing hypothermic and non-hypothermic groups in pediatric traumatic brain injury patients [22,24].

A total of two studies were included in a meta-analysis of cerebral perfusion pressure. The standard difference in means between hypothermic and non-hypothermic groups was 0.158 mmHg; however, the difference was insignificant (SMD = 0.158: 95% CI = 0.11–0.13, *p* = 0.172) (Figure 10).

**Figure 11:**
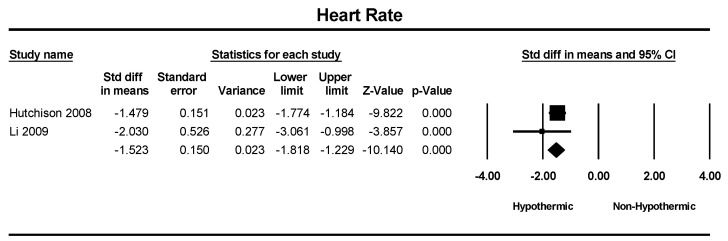
Forest plot depicting the meta-analysis of heart rate comparing hypothermic and non-hypothermic groups in pediatric traumatic brain injury patients [24,29].

Two studies were included in a meta-analysis of heart rate. The heart rate was significantly lower in the hypothermic group, with the standard difference in means of 1.523 (SMD = −1.523: 95% CI = −1.81–−1.22, *p* < 0.001) (Figure 11).

**Figure 12:**
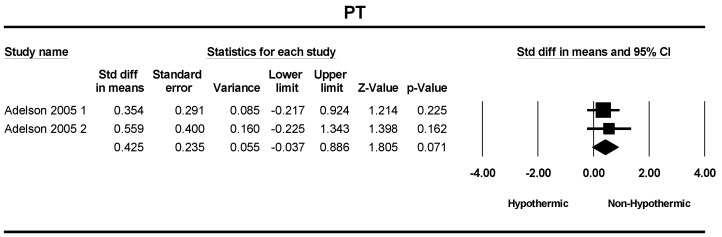
Forest plot depicting the meta-analysis of PT comparing hypothermic and non-hypothermic groups in pediatric traumatic brain injury patients [22].

**Figure 13:**
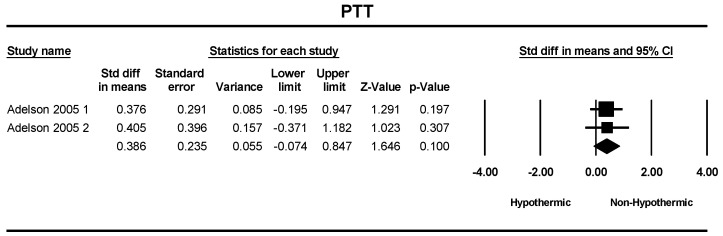
Forest plot depicting the meta-analysis of PTT comparing hypothermic and non-hypothermic groups in pediatric traumatic brain injury patients [22].

Two studies were included in a meta-analysis of PT and PTT. The standard differences in means of PT and PTT between the hypothermic and non-hypothermic groups were 0.425 (SMD = 0.425; 95% CI = −0.037–0.886, *p* = 0.07) and 0.386 (SMD = 0.386; 95% CI = −0.074–0.847, *p* = 0.10), respectively. The difference between groups was not statistically different (Figure 12 and Figure 13).

### 3.4. Sensitivity Analysis

A sensitivity analysis was conducted via leave-one-out analysis to evaluate the reliability and consistency of the meta-analysis results. The analysis indicated that the mortality outcomes were not robust, as *p* > 0.05 was observed in five out of seven analyses. Similarly, the results for infection and arrhythmia were found to lack robustness, as all studies showed *p* > 0.05. Regarding hypotension, the sensitivity analysis indicated that only one out of three studies showed *p* ≤ 0.05, suggesting that the meta-analysis results were not robust. In addition, the sensitivity analysis revealed that the outcomes for hospital and PICU length of stay, CPP, hemorrhage, PT, and PTT were not robust, as all studies showed *p* > 0.05. Conversely, the results for ICP and heart rate were deemed robust, as all studies showed *p* ≤ 0.05.

### 3.5. Publication Bias

**Figure 14:**
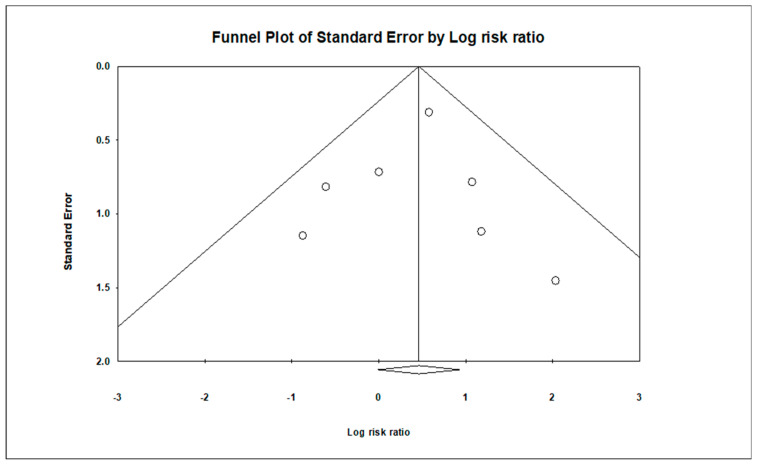
Funnel plot illustrating publication bias assessment for mortality, showing the distribution of effect sizes plotted against standard error. The symmetrical distribution indicates minimal publication bias.

**Figure 15:**
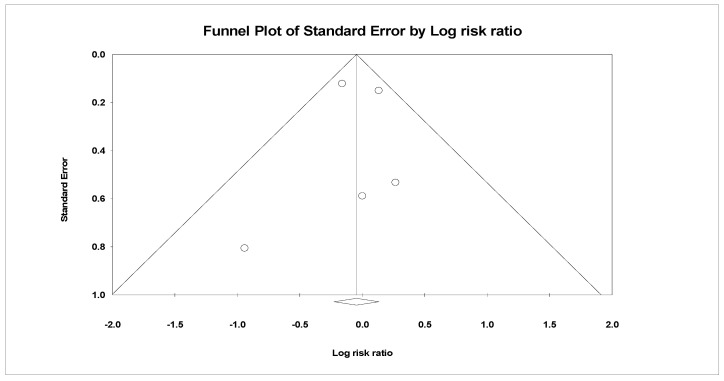
Funnel plot illustrating publication bias assessment for infection, showing the distribution of effect sizes plotted against standard error. The symmetrical distribution indicates minimal publication bias.

**Figure 16:**
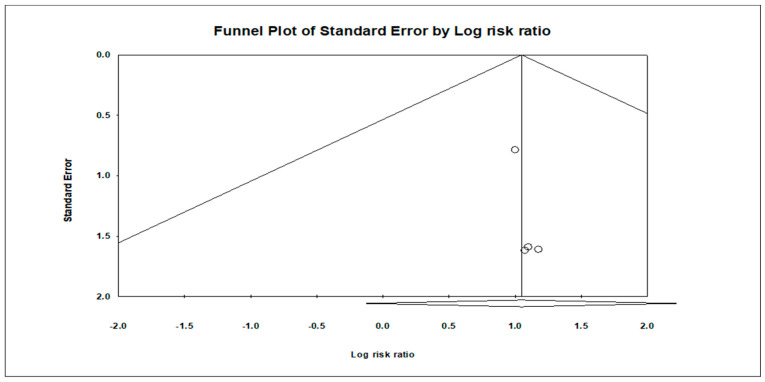
Funnel plot illustrating publication bias assessment for arrhythmia, showing the distribution of effect sizes plotted against standard error. The symmetrical distribution indicates minimal publication bias.

**Figure 17:**
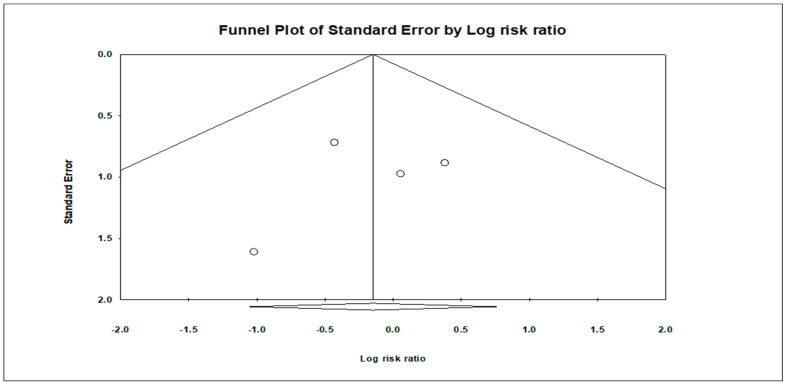
Funnel plot illustrating publication bias assessment for hemorrhage, showing the distribution of effect sizes plotted against standard error. The symmetrical distribution indicates minimal publication bias.

The analysis indicated insignificant publication bias for mortality (t = 0.16, *p* = 0.87), infection (t = 0.13, *p* = 0.90), arrhythmia (t = 2.89, *p* = 0.11), and hemorrhage (t = 0.419, *p* = 0.71). Funnel plots illustrating these findings are provided in Figure 14, Figure 15, Figure 16 and Figure 17.

## 4. Discussion

Following TBI, the body temperature is increased due to various cytokines being released. The higher temperature is attributed to the increase in the metabolism of CNS and the augmentation of cerebral blood flow, which leads to higher intracranial pressure if the compensatory mechanisms fail [30,31,32,33,34,35]. Moreover, it has been shown that a higher period of exposure to increased blood temperature will yield an increase in glutamate secretion, tissue damage, worsened cerebral ischemia, and eventually poor outcomes for the patients [35,36,37]. This injury to the brain makes it sensitive and vulnerable to the body temperature, and this sensitivity is even higher in children than adults. The head size in newborns and infants is disproportionately large and the head-to-body ratio is higher than in adults. In addition, the calvarium in children is thinner rather than in adults, and open sutures and fontanelles at early ages allow for additional damage following ICP changes, which leads to more diffuse injuries than that of adults [38,39]. 

The rationale for using therapeutic hypothermia in pediatrics with severe TBI is based on old experimental studies demonstrating that therapeutic hypothermia mitigates some of the secondary injury mechanisms instigated by TBI. Animal research has demonstrated that lowering body temperature by 1 °C decreases metabolism and cerebral blood flow by around 5–7%, thereby reducing oxygen consumption and carbon dioxide production [34,35]. In addition, experimental studies have demonstrated that mild post-traumatic hypothermia reduces the levels of excitatory neurotransmitters, proinflammatory cytokines, and lactate accumulation. This reduction in temperature not only decreases the need for anaerobic metabolism by lowering oxygen consumption but also leads to a decrease in membrane permeability [36,37,38,39,40,41,42]. Consequently, the influx of ions, particularly Ca^2+^ and Na^+^, is diminished, thereby reducing the cascade of events they initiate. Additionally, therapeutic hypothermia has been shown to prevent apoptosis by reducing the levels of caspase 3 and cytochrome C, which are mediators of cellular apoptosis and necrosis [42,43].

Despite promising findings in preclinical research, therapeutic hypothermia failed to demonstrate efficacy or safety when tested in clinical trials involving pediatric patients with severe TBI. Factors contributing to this translational/reproducibility crisis include the complexity of human biology, limited understanding of disease mechanisms, inadequate preclinical models, and variability in patient responses. Bridging this gap requires interdisciplinary collaboration, rigorous study design, improved translational research methods, and a deeper understanding of disease biology and patient heterogeneity. Addressing these challenges is essential to ensure that innovative therapies reach patients and improve clinical outcomes effectively [44].

In that light, our meta-analysis found that while hypothermia demonstrated neuroprotective effects in some aspects, its overall impact on mortality was not statistically significant. These findings are consistent with previous studies, suggesting that while hypothermia may offer neuroprotection, it may not translate into significant improvements in survival outcomes [20]. In line with our findings, a systematic review and meta-analysis by Zhang et al. [20] found no evidence supporting the efficacy of therapeutic hypothermia in treating children with TBI. It suggested that such treatment might elevate the risk of mortality and arrhythmia. The study failed to demonstrate any positive impact of therapeutic hypothermia on the prognosis of pediatric TBI patients, nor did it indicate an increased risk of pneumonia or coagulation dysfunction associated with this therapy. However, it is important to note that these findings were constrained by the quality of the studies included, warranting cautious interpretation. Similarly, in another meta-analysis, Crompton et al. [45] found therapeutic hypothermia to be a beneficial treatment option for adults following traumatic brain injuries, but its efficacy in children could not be endorsed based on the current evidence [45]. 

It has been reported that patients with TBI encounter a higher infection rate ranging from 50% to 70%, and are more susceptible to sepsis or pneumonia. It also has been noted that hypothermia decreases the leukocyte number in blood circulation, attributed to a higher risk of infection and mortality [46,47,48]; however, the infection rate was insignificantly lower in the hypothermia group.

Furthermore, our data revealed that patients in the hypothermia group had a slightly higher incidence of arrhythmia, although this difference was not statistically significant. This finding was in line with the results of Bourdagas et al. [49], who reported that five out of seven pediatric patients in the hypothermia group experienced arrhythmia following severe TBI. They also found that pediatric patients in the hypothermia group had a significantly lower heart rate, but did not experience bradycardia, which is consistent with our findings.

The occurrence of hypothermia in trauma patients has been a serious concern. It can lead to complications such as low platelet count, blood clotting problems, bleeding, shock, higher rates of hospital admission, and mortality, as well as the need for blood transfusions [50,51,52]. However, the results of the present study indicate that the therapeutic hypothermia method did not have a significant impact on the length of hospital/PICU stay, the occurrence of bleeding, PT, and PTT when compared to normothermia patients following TBI. However, some papers have concluded that the initial 24 h therapeutic hypothermia increases the mortality rate in both children and adults [24,53]. 

The difference seen between the response of adult patients with TBI (which is promising) with pediatric patients to therapeutic hypothermia might be due to several reasons, as stated in the meta-analysis of Crompton et al. They showed that using hypothermia in adults following TBI is more beneficial than in children. However, children of older ages might benefit from hypothermia treatment due to behaving as adults based on metabolism and energy expenditure [45].

Children often respond differently to injury due to their distinct physiology and metabolic differences from adults, which may explain their less favorable outcomes [22]. Following TBI, children typically exhibit only 70% of their normal energy expenditure levels compared to adults, who often display a hypermetabolic response [54,55]. As hypothermia aims to reduce metabolic response [56], the significantly lower energy levels in children may provide a diminished target for hypothermic intervention, thereby limiting its potential to improve outcomes.

To summarize the results from other studies, it has been shown that a slightly lower mortality rate, lower infection rate, higher incidence of hypotension, lower occurrence of hemorrhage, higher incidence of arrhythmias, and CPP, PT, and PTT differences occurred in the non-hypothermic group compared to the hypothermic group, which was not statistically significant. Moreover, the difference in mean values of hospital stay duration was 0.10 days and 0.4 days for PICU stay, which was not statistically significant. However, heart rate was significantly lower in the hypothermic group rather than the control group.

While our meta-analysis provides valuable insights into the efficacy and safety of therapeutic hypothermia in pediatric TBI, several limitations should be acknowledged. First, the included studies varied in their methodological rigor and patient populations, which may have introduced heterogeneity and bias into the analysis. For example, the included studies may have utilized varying protocols for therapeutic hypothermia, including differences in target temperature, duration of cooling, and rewarming strategies. This variability could introduce heterogeneity into the analysis and impact the interpretation of the results. Also, the patient populations across the included studies may have differed in terms of age, comorbidities, and pre-existing conditions. Variability in patient characteristics could influence treatment response and contribute to heterogeneity in the analysis. Some studies may have lacked comprehensive reporting of outcomes, including incomplete data on adverse events, patient outcomes, and follow-up assessments. Incomplete reporting of data could limit the accuracy and reliability of the meta-analysis results. Additionally, the sample sizes of some studies were relatively small, limiting the statistical power of the analysis. Moreover, the optimal duration and depth of hypothermia therapy remain unclear, and individual patient characteristics may influence treatment response. Further well-designed, randomized controlled trials are warranted to elucidate the true efficacy and safety profile of therapeutic hypothermia in this vulnerable patient population. Such trials should consider standardizing treatment protocols, optimizing patient selection criteria, and incorporating long-term outcome assessments to provide more robust evidence for guiding clinical decision-making in the management of pediatric severe TBI. Until then, clinicians should approach the use of therapeutic hypothermia in pediatric severe TBI patients with caution, recognizing the need for further research to clarify its role as a therapeutic intervention.

## 5. Conclusions

While therapeutic hypothermia has been proposed as a potential neuroprotective intervention for pediatric patients with severe TBI, our meta-analysis suggests its uncertain therapeutic efficacy. Despite preclinical evidence indicating neuroprotective benefits, our findings indicate that hypothermia did not significantly improve key clinical outcomes in these patients. Moreover, the observed reduction in heart rate, although statistically significant, may reflect a physiological response rather than a true therapeutic effect. These results underscore the need for a cautious interpretation of the therapeutic potential of hypothermia in this population.

## Figures and Tables

**Table 1:** Demographic characteristics.

First Author Name	Country	Type of Study	Number of Patients in Hypothermia Group	Number of Patients in Control Group	Male (Hypothermia Group)	Male (Control Group)	Female (Hypothermia Group)	Female (Control Group)	Mean Age (Hypothermy Group)	SD Age (Hypothermia Group)	Mean Age (Control Group)	SD Age (Control Group)	Follow-Up Duration
Adelson 2005 [22]	US	Phase II multi-center, randomized, controlled trial	23	25	27		21		6.92	3.09	6.86	3.81	3 to 6 months
			13	13	17		9		7.17	6.64	5.6	5.23	
Adelson 2013 [23]	US	Phase 3, multi-center, multinational, randomized controlled trial	39	38	21 (54%)	27 (71%)	18 (46%)	11 (29%)	9·7	(4.2–14.5)	12.5	(3.3–14.8)	3 MONTHS
Hutchison 2008 [24]	US	Multicenter, international trial	108	117	70 (65%)	71 (61%)	38 (35%)	46 (39%)	9.8	4.9	10.2	4.8	6 MONTHS
Beca 2015 [25]	Australia, New Zealand	Multi-center prospective randomized controlled phase II trial	24	26	11 (46%)	16 (62%)	13 (54%)	10 (38%)	11	(6.9–14.2)	9.5	(5.2–13.8)	12 MONTHS
Hutchison 2006 [26]	Canada	RCT	39	0	29 (74.4%)	0	10 (25.6%)	0	N/A	N/A	0	0	6 months
Biswas 2002 [27]	US	RCT	10	11	N/A	N/A	N/A	N/A	5.9	2.9	6.6	3.7	12 MONTHS
Grinkeviciute 2009 [28]	Lithuania	Prospective, observational, clinical trial	8	0	4 (50%)	0	4 (50%)	0	10.7	6.4	0	0	24 months
Li 2009 [29]	China	RCT	12	10	N/A	N/A	N/A	N/A	8.7 months	8–96 months	11.4 months	6–108 months	N/A

N/A: not applicable.

**Table 2:** TBI treatment.

First Author Name	Severity of TBI	Mechanisms of Injury (Fall)	Mechanisms of Injury (Motor Vehicle Collision)	Mechanisms of Injury (Recreational Injury)	Mechanisms of Injury (Assault)	Location of Body Temperature Measurement	Control Group Temperature Degree	Hypothermia Therapy Degree	Duration of Hypothermia Therapy	GCS Score at Admission [Control]	GCS Score at Admission [Hypo]	Time Interval between Injury and Cooling [HOURS]	Time Interval between Injury and Targeted Temperature
Adelson 2005 [22]	Glasgow Coma Scale score < 8	N/A	N/A	N/A	N/A	rectal	36.5 to 37.5 °C	32–33 °C	48 h	5.64 ± 1.63	5.74 ± 1.39	4.62 ± 1.09	9.57 ± 2.81
	Glasgow Coma Scale score < 8	N/A	N/A	N/A	N/A					6.23 ± 1.2	6.42 ± 1.2	15.03 ± 7.08	20.78 ± 6.92
Adelson 2013 [23]	Glasgow Coma Scale score < 8	13 (17%)	51 (66%)	N/A	1 (1%)	rectal or brain temperature probes	36·5–37.5 °C	32–33 °C	48 h	6 (5–7)	6 (5–7)	5 h 8 min (SD 55 min)	3 h 10 min (4 h 31 min to 22 h 18 min) [Time to cool]
Hutchison 2008 [24]	Glasgow Coma Scale score < 8	38	134	N/A	N/A	esophagus	36.9 ± 0.5 °C	33.1 ± 1.2 °C	24 h	5 (3–6)	5 (4–6)	6.3 ± 2.3	3.9 ± 2.6 h [Time to cool]
Beca 2015 [25]	Glasgow Coma Scale score < 8	N/A	N/A	N/A	N/A	esophagus	36–37 °C	32–33 °C	72 h	4.5 (3–7)	5.5 (3.5–7)	5.3 (4.4–6.0) h	9.3 h (8.0–10.9 h)
Hutchison 2006 [26]	Glasgow Coma Scale score < 8	4 (10.35%)	28	N/A	N/A	esophagus	0	32–33 °C	24 h	0	5.18 ± 1.6	6.48 ± 2.3	3.88 ± 2.5 [Time to cool]
Biswas 2002 [27]	Glasgow Coma Scale score < 8	0	19	0	0	rectal	36.5–37.5 °C	32–34 °C	48 h	5.6 (3–7)	4.7 (3–8)	246 ± 86 min [HYPO], 267 ± 57 min [NORMO]	N/A
Grinkeviciute 2009 [28]	Glasgow Coma Scale score < 8	1	7	0	0	retal	0	33–34 °C	48 h	0	7 (4–8)	6	8–9
Li 2009 [29]	Glasgow Coma Scale score < 8	7	12	3	0	rectal	38.0 ± 0.5	34.5 ± 0.28 °C	72 h	6.5 ± 1.7	6.4 ± 1.8	7.2 ± 1.4 h	N/A

**Table 3:** Outcome of hypothermia group.

First Author Name	Mortality Rate (n, %)	Hypotension	Coagulopathy Rate (n, %)	Infection Rate (n, %)	Arrhythmia Rate (n, %)	Early Intracerebral Hemorrhage (n, %)	Late Intracerebral Hemorrhage (n, %)	Mean ICP (Early)	ICP (SD)	Mean CPP (Early)	CPP (SD)	PT (Mean)	PT (SD)	PTT (Mean)	PTT (SD)	Heart Rate (Mean)	Heart Rate (SD)	Pediatric Intensive Care Unit (PICU) Length of Stay [DAY]	PICU Length of Stay (SD)	Hospital Length of Stay (Mean) [DAY]	Hospital Length of Stay (SD)	Score Final	
																						GOOD	POOR
Adelson 2005 [22]	2 (8%)	N/A	7	6	5	2		16.57	18.73	66.3	14.41	14.09	1.98	30.5	3.37	N/A	N/A	N/A	N/A	N/A	N/A	14	18
	3 (24%)	N/A		4	1							13.81	1.64	30.24	2.43	N/A	N/A	N/A	N/A	N/A	N/A		
Adelson 2013 [23]	6 (18%)	3 (9%)	29 (37%)	2 (6%)	1 (3%)	3 (9%)		N/A	N/A	N/A	N/A	N/A	N/A	N/A	N/A	N/A	N/A	N/A	N/A	N/A	N/A	22 (58%)	16 (42%)
Hutchison 2008 [24]	23 (21%)	49 (45%)	N/A	39 [Pneumonia], 16 [Other infections]	N/A	N/A	3 (3%)	14.7	10.7	66.4	12	N/A	N/A	N/A	N/A	81.5	16.7	11.5	7.1	30.2	31.7	N/A	N/A
Beca 2015 [25]	3 (13%)	4	N/A	6 [Pneumonia], 1 [Urinary tract], 3 [Wound], 10 [Other]	1 (4%)	0	0	12.5		67.6		N/A	N/A	N/A	N/A	95		12	8–15	48	28–77	N/A	4 (17%)
Hutchison 2006 [26]	8 (20.5%)	N/A	N/A	16 [Pneumonia], 1 [Septic shock], 6 [Other infections]	1 (2.6) [Ventricular tachycardia]	5 (12.8%)		15.2	10.1	69.9	10.8	13	4.3	34.8	11.3	88.9	20.4	N/A	N/A	N/A	N/A	22 (62.9%)	N/A
Biswas 2002 [27]	3	N/A	N/A	N/A	N/A	0	0	10		27		N/A	N/A	N/A	N/A	N/A	N/A	N/A	N/A	N/A	N/A	6	4
Grinkeviciute 2009 [28]	0	0	0	0	0	0	0	N/A	N/A	N/A	N/A	N/A	N/A	N/A	N/A	N/A	N/A	11.2	5	16	5.6	8	0
Li 2009 [29]	1 (8.3%)	N/A	N/A	N/A	N/A	N/A	N/A	12.45	1.03	N/A	N/A	N/A	N/A	N/A	N/A	109.92	19.48	N/A	N/A	N/A	N/A	N/A	N/A

**Table 4:** Outcome of control group.

First Author Name	Mortality Rate (n, %)	Hypotension	Coagulopathy Rate (n, %)	Infection Rate (n, %)	Arrhythmia Rate (n, %)	Early Intracerebral Hemorrhage (n, %)	Late Intracerebral Hemorrhage (n, %)	Mean ICP (Early)	ICP (SD)	Mean CPP (Early)	CPP (SD)	PT (Mean)	PT (SD)	PTT (Mean)	PTT (SD)	Heart Rate (Mean)	Heart Rate (SD)	PICU Length of Stay	PICU Length of Stay (SD)	Hospital Length of Stay (Mean)	Hospital Length of Stay (SD)	GCS Score Final	
																						GOOD	POOR
Adelson 2005 [22]	4 (16%)	N/A	6	5	2	2		17.88	17.25	64.8	16.58	13.51	1.25	29.17	3.68	N/A	N/A	N/A	N/A	N/A	N/A	14	22
	3 (24%)			4	0							12.95	1.43	28.83	4.28	N/A	N/A	N/A	N/A	N/A	N/A		
Adelson 2013 [23]	2 (5%)	1 (2%)	24 (49%)	5 (12%)	0	2 (5%)		N/A	N/A	N/A	N/A	N/A	N/A	N/A	N/A	N/A	N/A	N/A	N/A	N/A	N/A	22 (58%)	16 (42%)
Hutchison 2008 [24]	14 (12%)	38 (32%)	N/A	51 [Pneumonia], 19 [Other infections]	N/A	N/A	5 (4%)	17.1	11.1	64.3	11.5	N/A	NN	N/A	N/A	108.1	19.1	11.3	7.2	28.3	24.2	N/A	N/A
Beca 2015 [25]	1 (4%)	0	N/A	7 [Pneumonia], 2 [Urinary tract], 3 [Wound], 7 [Other]	0	1 (4%)		13		62.5		N/A	N/A	N/A	N/A	97		11	8–14	40	27–60	N/A	3 (12%)
Hutchison 2006 [26]	0	0	0	0	0	0	0	0	0	0	0	0	0	0	0	0	0	0	0	0	0	0	0
Biswas 2002 [27]	0	N/A	N/A	N/A	N/A	0	0	10.4		38.8		N/A	N/A	N/A	N/A	N/A	N/A	N/A	N/A	N/A	N/A	11	0
Grinkeviciute 2009 [28]	0	0	0	0	0	0	0	0	0	0	0	0	0	0	0	0	0	0	0	0	0	0	0
Li 2009 [29]	2 (20%)	N/A	N/A	N/A	N/A	N/A	N/A	25.62	1.55	N/A	N/A	N/A	N/A	N/A	N/A	153.7	23.88	N/A	N/A	N/A	N/A	N/A	N/A
